# Trained facilitators’ experiences with structured advance care planning conversations in oncology: an international focus group study within the ACTION trial

**DOI:** 10.1186/s12885-019-6170-7

**Published:** 2019-10-31

**Authors:** M. Zwakman, K. Pollock, F. Bulli, G. Caswell, B. Červ, J. J. M. van Delden, L. Deliens, A. van der Heide, L. J. Jabbarian, H. Koba-Čeh, U. Lunder, G. Miccinesi, C. A. Møller Arnfeldt, J. Seymour, A. Toccafondi, M. N. Verkissen, M. C. Kars, On behalf of the ACTION consortium, A. van der Heide, I. J. Korfage, J. A. C. Rietjens, L. J. Jabbarian, S. Polinder, P. F. A. Billekens, J. J. M. van Delden, M. C. Kars, M. Zwakman, L. Deliens, M. Verkissen, K. Eecloo, K. Faes, K. Pollock, J. Seymour, G. Caswell, A. Wilcock, L. Bramley, S. Payne, N. Preston, L. Dunleavy, E. Sowerby, G. Miccinesi, F. Bulli, F. Ingravallo, G. Carreras, A. Toccafondi, G. Gorini, U. Lunder, B. Červ, A. Simonič, A. Mimić, H. Kodba Čeh, P. Ozbič, M. Groenvold, C. Møller Arnfeldt, A. Thit Johnsen

**Affiliations:** 1 Julius Center for Health Sciences and Primary Care, University Medical Center Utrecht, Utrecht, the Netherlands; 2 Faculty of Medicine and Health Sciences, School of Health Sciences, University of Nottingham, Nottingham, UK; 3 Oncological network, research and prevention Institute – ISPRO, Florence, Italy; 4 University Clinic for Respiratory and Allergic Diseases Golnik, Golnik, Slovenia; 5 End-of-life Care Research Group, Vrije Universiteit Brussel, Brussels, Belgium; 6 Department of Public Health and Primary Care, Ghent University, Ghent, Belgium; 7 Department of Public Health, Erasmus MC, University Medical Center Rotterdam, Rotterdam, the Netherlands; 8 Department of Public Health, University of Copenhagen, Copenhagen, Denmark; 9 Department of Palliative Medicine, Bispebjerg and Frederiksberg Hospital, The Research Unit, Copenhagen, Denmark; 10 University of Sheffield, Sheffield, UK; 11 Ghent University, Brussels, Belgium

**Keywords:** Advance care planning, Facilitator, Respecting choices, Experiences, Cancer

## Abstract

**Background:**

In oncology, Health Care Professionals often experience conducting Advance Care Planning (ACP) conversations as difficult and are hesitant to start them. A structured approach could help to overcome this. In the ACTION trial, a Phase III multi-center cluster-randomized clinical trial in six European countries (Belgium, Denmark, Italy, the Netherlands, Slovenia, United Kingdom), patients with advanced lung or colorectal cancer are invited to have one or two structured ACP conversations with a trained facilitator. It is unclear how trained facilitators experience conducting structured ACP conversations. This study aims to understand how facilitators experience delivering the ACTION Respecting Choices (RC) ACP conversation.

**Methods:**

A qualitative study involving focus groups with RC facilitators. Focus group interviews were recorded, transcribed, anonymized, translated into English, and thematically analysed, supported by NVivo 11. The international research team was involved in data analysis from initial coding and discussion towards final themes.

**Results:**

Seven focus groups were conducted, involving 28 of in total 39 trained facilitators, with different professional backgrounds from all participating countries. Alongside some cultural differences, six themes were identified. These reflect that most facilitators welcomed the opportunity to participate in the ACTION trial, seeing it as a means of learning new skills in an important area. The RC script was seen as supportive to ask questions, including those perceived as difficult to ask, but was also experienced as a barrier to a spontaneous conversation. Facilitators noticed that most patients were positive about their ACTION RC ACP conversation, which had prompted them to become aware of their wishes and to share these with others. The facilitators observed that it took patients substantial effort to have these conversations. In response, facilitators took responsibility for enabling patients to experience a conversation from which they could benefit. Facilitators emphasized the need for training, support and advanced communication skills to be able to work with the script.

**Conclusions:**

Facilitators experienced benefits and challenges in conducting scripted ACP conversations. They mentioned the importance of being skilled and experienced in carrying out ACP conversations in order to be able to explore the patients’ preferences while staying attuned to patients’ needs.

**Trial registration:**

International Standard Randomised Controlled Trial Number registry 63110516 (ISRCTN63110516) per 10/3/2014.

## Background

Advance Care Planning (ACP) is a process of conversations with patients about their values, goals and preferences for future medical treatment and care and has the potential to improve the quality of end of life care [1–3].

Previous studies report that, due to a lack of knowledge and experience in how to initiate and facilitate ACP conversations, many health care professionals (HCPs) have difficulty conducting ACP conversations [4–10]. The fear of harming the patient’s coping strategies or damaging their professional relationship with the patient are also important barriers to HCPs initiating an ACP conversation [4–8, 10]. A structured approach and delivery by trained facilitators could be strategies to overcome these barriers, thus facilitating ACP in clinical practice [11, 12]. Until now, research has focussed on the patients’ experiences who participated in a structured ACP conversation [13]. It has not been investigated yet how trained facilitators experience the use of a structured approach and whether this could, in their view, resolve some of the reported barriers to carrying out ACP conversations.

Currently, there are many different approaches to carrying out ACP in different settings [1]. One of the most well-known ACP programmes is the Respecting Choices (RC) ACP programme [14, 15]. Since its initiation in 1993, the RC ACP programme has developed towards a structured and widely used programme, particularly in the USA [16–18]. An adapted version of the RC ACP programme was tested in the ACTION trial [19]. The ACTION trial was a Phase III multi-centre cluster-randomised clinical trial carried out in six European countries (Belgium (BE), Denmark (DK), Italy (IT), the Netherlands (NL), Slovenia (SI) and the United Kingdom (UK)) (see Additional file 1). The ACTION RC ACP intervention involved one or two scripted conversations between an ACTION RC ACP trained facilitator, a patient diagnosed with advanced lung- or colorectal cancer and, if the patient wished, a person nominated as their personal representative (PR). Attending the ACTION RC ACP conversation, enabled the PR to understand the role of PR, to become familiar with the patient’s views and wishes and encouraged an open dialogue between the patient and the PR. The facilitators assisted patients during the ACTION ACP RC conversations in exploring their understanding of their illness, reflecting on their goals, values and beliefs, and to consider their future treatment preferences and decisions. Facilitators also informed patients about the opportunity to document their preferences for (future) medical treatment and care in the so-called My Preferences form (see Additional file 2) [19].

This paper presents findings from a qualitative study which was part of the ACTION trial. The study aimed at exploring the ACTION RC facilitators experiences with carrying out the structured RC ACP conversations with patients and their relatives and whether this could overcome barriers to conduct an ACP conversation.

## Method

### Research design

To get insight into the ACTION RC ACP facilitators’ experiences, focus groups were undertaken in each of the participating countries and thematically analysed [20]. The study is reported following the Consolidated Criteria for Reporting Qualitative Research (COREQ) Guidelines [21].

### Participants

To become a facilitator in the ACTION study, respondents had to have working experience as a HCP in care for patients with cancer (e.g. as a nurse, doctor or social worker) and were willing to deliver the intervention as part of their job. To participate in the ACTION study, facilitators had to take a two-day ACTION RC ACP training, that consisted of role plays, videos demonstrating RC ACP conversations and homework assignments (see also Additional file 2). Facilitators were eligible for participation in the focus group if they had undertaken an ACTION RC ACP conversation with at least three patients, to ensure that they had gained some experience with the delivery of the ACTION RC ACP conversations. Eligible facilitators were invited by email.

### Data collection

In the summer of 2016 we conducted one focus group in each participating country. Each focus group lasted approximately 1.5 h, and was carried out in a private room in the hospital where the facilitators worked. Personal background information was collected before the start of the focus group. An aide memoire, consisting of open-ended questions and a set of prompts for each question, was used to guide the focus groups. This aide memoire, based on literature and expert knowledge of the multidisciplinary international ACTION research team, covered four main topics: (1) prior experience with conducting ACP conversations, (2) prior thoughts about the ACTION RC ACP intervention, (3) experiences with the ACTION RC ACP training and (4) experiences with conducting the ACTION RC ACP conversations (Table 1). All focus groups were moderated and observed by one or two male and female qualitative researchers involved in the ACTION trial with a background either in health science, psychology, psychiatry, anthropology or nursing. They ensured that all predefined topics were discussed and made field notes during the focus group. Some moderators knew the participants before the start of the focus group, for example because the moderator had also been present at the ACTION RC training. All focus groups were recorded and transcribed verbatim.

**Table 1:** Facilitator focus group aide memoire

Main topics	Prompts
Understanding of ACP before ACTION	What was your experience of ACP before the ACTION trial?
Experience of ACTION and RC ACP intervention	What were your initial thoughts about the ACTION RC ACP intervention?
Experience of RC ACP intervention training	- How would you assess the training you received about the ACTION RC ACP intervention and how to discuss this with patients? - How helpful was the training in enabling you to feel confident about delivering the ACTION RC ACP intervention?
Experience of delivering the ACTION RC ACP conversations	- Can you tell us about your experience of delivering the ACTION RC ACP intervention? Was having a standard script helpful/unhelpful? - How did you feel about the support you received? - How did patients and Personal representatives respond? - Will you/have you used the RC approach, or aspects of it, in your normal practice (outside the ACTION trial)? - Were there any things you found difficult or challenging? - Do you think patients found it helpful or distressing?

Abbreviations: *RC* Respecting Choice, *ACP* Advance Care Planning

### Data analysis

**Fig. 1:**
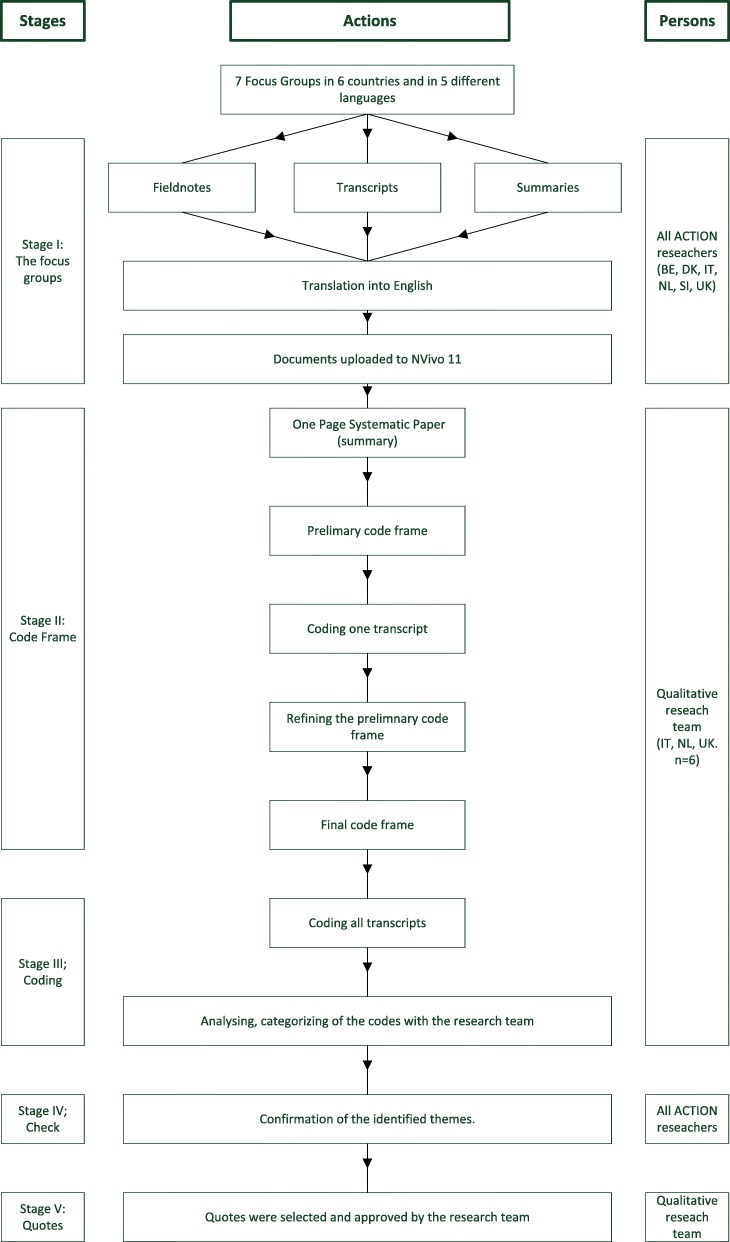
Process data analysis

Thematic analysis was based on the stepwise approach of the Qualitative Analysis Guide of Leuven (QUAGOL) [20]. This guide was adjusted by the international qualitative research team (MZ, MK, AT, FB, GM, GC, KP) to accommodate the international scope of this study. A detailed description of the steps taken is visualised in Fig. 1.

During the first stage, the transcriptions were anonymized, translated into English and uploaded to NVivo 11. In stage two, each member of the international qualitative research team wrote a summary of the key storylines of all focus group interviews. Based on these summaries, a preliminary coding framework including a description of the content of each code was developed (MZ). The members of the qualitative research team tested and developed the coding framework by independently coding the same focus group transcript. The team discussed the coded transcripts during several meetings until arriving at a consensus on definitions and application of codes and sub codes (Table 2).

**Table 2:** Coding framework

Main codes	Subcodes
Prior experiences with ACP	
Thoughts about ACTION	
Reasons to participate in ACTION	
Becoming a facilitator	The RC training Support during the study Learning by doing Personal and professional growth Becoming aware of RC
Cultural issues	During the training During the conversations
Aspects RC	Script_positive Script_helpful questions Script_negative Script_difficult questions Script_lay-out My preferences form
Preconditions RC	Timing Place of the conversation
Being a facilitator	Needed skills Dual role facilitator Be involved in the regular care Not involved in the regular care Out of their comfort zone Workload Uncertainty Responsibility
Impressions concerning patients	Reasons for patients to participate Investment Preparation Difficulties Patients responses The fit between RC and the patient
Personal representative	Awareness of their role Influence on the conversation
The value of ACTION RC ACP conversations	Opportunity to reflect and talk Empowerment of patients Quality of life Relationship patient-facilitator Communication patient-PR Patients undertake actions Have the time to conduct an ACP conversation Helpful
Impact on current practice	Using the intervention Managing study and daily practice
ACP in the future	Fit RC intervention to patients Setting Script Part of routine job Risks for the future Improvements Implementation of the intervention
Being part of a research	The feeling of being watched by the researcher Wanted to do it right Patients should benefit from it Use as an excuse to the questions they ask

Abbreviations: *RC* Respecting Choice*, ACP* Advance Care Planning

The first researcher (MZ) coded all transcripts in the third stage. To ensure the validity of the coding process, each transcript was also independently coded by a second researcher of the qualitative research team. After each coded transcript, discrepancies regarding coding were solved during telephone meetings and the content of the transcript was discussed. Subsequently, codes were categorised and themes were identified. This process was supported by the development of mind maps (MZ, MK) and validated by the qualitative research team. Saturation was achieved, meaning that the analysis of the last two focus group interviews did not uncover ideas that could not be assigned to already existing themes [22].

In stage four, all researchers who had attended one of the focus groups checked and approved the identified themes.

In the final stage, relevant quotes to illustrate the identified themes were extracted by MZ and approved by the qualitative research team.

### Ethical consideration

Ethical approval for the ACTION trial, including the qualitative work package, was obtained from the locally responsible Research Ethics Committees in all countries and institutions. Written informed consent was obtained from all participating facilitators.

## Results

### Participant demographics

We conducted seven facilitator focus groups in six participating countries (for logistic reasons Dutch facilitators were split into two focus groups). Of the 39 facilitators involved in the ACTION trial, 28 participated in the focus group interviews. One facilitator (SI) had conducted only one conversation and was erroneously included (Table 3). Eight of the 11 excluded facilitators had undertaken less than three ACTION RC ACP conversations. The 28 included facilitators had conducted ACTION RC ACP conversations with six patients on average, ranging from one to 14 patients.

**Table 3:** Facilitators per country

Country	Number of trained facilitators within the ACTION trial	Respondents n (%)	Reasons to not included	The number of respondents involved in the clinical care for some of the patient’s n (%)
BE	10	4 (40%)	*n* = 5: performed less than 3 ACTION RC ACP conversations *n* = 1: not able to participate in the FG	1 (25%)
DK	4	4 (100%)	n.a.	3 (75%)
IT	7	4 (57,1%)	*n* = 3: performed less than 3 ACTION RC ACP conversations	4 (100%)
NL	8	7 (87,5%)	*n* = 1: Logistic reasons (time and availably)	5 (71,4%)
SI	5	5 (100%)	n.a.	0 (0%)
UK	5	4 (87,5%)	*n* = 1: Logistic reasons (time and availably)	0 (0%)
Total	39	28 (71,8%)		13 (46,4%)

Abbreviations: *FG* Focus Group, *ACTION RC ACP* ACTION Respecting Choice Advance Care Planning

Most facilitators were female (*n* = 24), HCPs (*n* = 22) and in most cases a nurse (*n* = 18). Eighteen facilitators had during their career participated in a palliative care course. Thirteen of the 22 HCP-facilitators were involved in clinical care of patients to whom they had delivered the ACTION RC ACP conversations (Tables 3 and 4). For each citation below it is indicated whether the facilitator was involved in the care of the patient or not.

**Table 4:** Facilitator background information

	Facilitator n (%) or mean (range) *n* = 28
Age	44 years (28 – 58)
Gender	
Male	4 (14.3%)
Female	24 (85.7%)
Highest educational qualification	
Doctoral or equivalent	4 (14.3%)
Master degree or equivalent	9 (32.1%)
University degree or equivalent	8 (28.6%)
Post-secondary, non-tertiary	6 (17.9%)
Not elsewhere classified; finishing a master degree	1 (3.6%)
Education: palliative care course	
Yes	18 (64.3%)
No	10 (35.7%)
Current professional role	
Health Care professional	22 (78.6%)
Nurse	8 (28.6%)
Nurse coordinator	1 (3.6%)
Nurse specialist (in training)	9 (32.1%)
Oncologist	1 (3.6%)
Social worker	1 (3.6%)
Clinical psychologist	2 (7.2%)
No Health Care professional	6 (21.4%)
Researcher	3 (10.7%)
Senior consultant	1 (3.6%)
Lead hospital unit	2 (7.2%)
Involvement in the care for ACTION patients	
Yes	8 (28.6%)
For some patients	5 (17.9%)
No	15 (53.6%)
Work experience	20.2 years (4 – 36)

### Themes

From the experiences of facilitators delivering the ACTION RC ACP conversations six themes were identified; (1) A welcomed opportunity, but challenging, (2) Experiences with using the script, (3) Helpful and difficult, (4) Feeling uncertain and responsible, (5) Learning process, and (6) Thoughts about implementation. Below we describe these themes in detail.

### A welcomed opportunity, but challenging

The facilitators’ experiences with ACP, prior to their participation in the ACTION trial, were diverse. Four facilitators appeared to be skilled and clinically experienced in a more comprehensive type of ACP conversations, the so-called ‘family conversations’. Three facilitators were familiar with the concept of ACP, but had no clinical experience with it. However, the majority of facilitators (*n* = 22) were involved in clinical practice and were used to discussing particular aspects of ACP, such as the preferred place of care, cardiopulmonary resuscitation or palliative sedation. Most described discussing these topics in an ad hoc and unstructured manner, usually in response to patient cues and fine-tuned to the patient’s coping style. When these topics were discussed, it was usually when the patient had reached an advanced stage of illness.

Based on clinical experience and previous understanding, many facilitators had a positive disposition towards ACP. They believed that ACP conversations were a suitable answer to the needs they perceived among patients with advanced cancer.

*‘I personally think that it is a very important thing [ACP] and I am very aware of its importance, working with our patients. Being able to speak about how to deal with care and also the end, in essence, of life, is a fundamental aspect’* (IT, HCP, involved).

In anticipation of their participation in the ACTION study, most facilitators welcomed the opportunity to become a facilitator. They considered participation in the ACTION trial to be an opportunity to learn new skills. They expected that the ACTION trial could contribute to the normalisation of ACP as a routine part of care and could support them to discuss difficult topics.

In addition to their positive stance towards becoming a facilitator, participants also anticipated some challenges. The majority of the facilitators expected the conversations to be difficult. In particular, facilitators without medical expertise feared being confronted with medical questions. Others thought that working with a script would require great changes to their normal ways of communicating with patients, and as such would be demanding. Lastly, some facilitators had doubts about the appropriateness of the ACTION ACP RC conversations for some of the patients, because the treatment of lung cancer stage 3a and 3b is often aimed to be curative.

*‘I had this feeling [of wrong timing] in advance, I thought: then we are going to say to those people [patients with lung cancer stage 3a and 3b] that we will give a treatment aimed at cure, and then we come up with this ACTION study’* (NL, HCP, involved).

### Experiences with using the script

In the ACTION trial, facilitators had dedicated time to schedule ACTION appointments with patients and were asked to carry out the RC ACP conversations according to a script. Facilitators who positively valued the scripted approach mentioned that it enabled them to conduct ACP in a more structured and comprehensive manner than they were used to. The script also offered support in which topics could be addressed in ACP and helped them to ask questions they perceived to be difficult for patients to cope with. Some questions of the script were especially positively valued. For example, the question ‘If you were having a good day, what would happen on that day?’ was experienced as a key topic that revealed a lot of relevant information about how patients lived and coped with their illness. Because of this, several facilitators had already started to use their experiences from involvement in the ACTION study in their wider practice.

*‘ …and it [the script] is helpful with questions about hope and… about pushing through, asking for prior experiences, these are points that the script covers very well’* (NL, HCP, involved).

Although facilitators evaluated the script as helpful at times, most also felt frustrated by the structured approach of the conversation. This was caused by their sense of being forced to follow the script even when they thought that topics were not presented in what they believed to be the right order, or to ask questions that they considered inappropriate for the category of patients under study, particularly in relation to patients’ illness process and well-being. Consequently, facilitators felt they risked losing rapport and becoming less aligned with patients.

‘*That heart and mind clash at such a moment’* (NL, HCP, not involved).

*‘The topics are not impossible… but the guide is impossible’* (DK, no HCP, involved).

In particular, facilitators who were not involved in regular patient care and, consequently, did not have a prior relationship with patients, found that the formality and structure of the script could hamper creating a trusting relationship with patients during the ACTION RC ACP conversation. Facilitators who worked in clinical practice had already developed their own style of communication with severely ill patients. Working in accordance with the script forced them to use different (e.g. more medically-orientated) language compared to what they were used to and to ask ACP-related questions they would not otherwise have asked. This took many facilitators outside their comfort zone. They described it as a major challenge to balance working with the script and having a meaningful and sensitive discussion with the patients and their PRs.

Some variance between the six participating countries in terms of facilitators’ experiences with specific questions was encountered. For some facilitators the questions about hope (‘*What do you hope for with your current medical plan of care*?’ followed by ‘*If all these hopes do not come true, what else would you hope for?*’) were difficult to ask because they did not want to distress patients. The Italian facilitators, in particular, felt uncomfortable asking what patients would hope in case the hopes for current medical treatment would not come true, because, from their perspective, this involved a risk of taking away the patients’ hope. In contrast, several facilitators from other countries felt positive about the questions regarding hope. They mentioned that, although challenging, these questions led to an in-depth understanding of patients’ ideas and views regarding their future in relation to the expected course of their illness.

*‘I think it [hope question] sometimes turns out to be crucial, to get people to open up’* (SI, HCP, not involved).

### Helpful aspects and difficulties with the structured ACP conversation

When undertaking ACTION RC ACP conversations, facilitators did not only experience what it was like to conduct these conversations, but also observed the responses of the patients and PRs involved in the conversations. Facilitators concluded that most patients were positive about having had an ACTION RC ACP conversation, which was encouraging to them. Facilitators reported that some patients spontaneously shared their positive feelings subsequent to the conversation. Patients told them they appreciated the information received or were grateful for being given the opportunity to discuss perspectives and preferences for future care and treatment they had not thought about before. One patient for instance, after having been transferred to a hospice, contacted the facilitator to say, ‘thank you’. ‘*It was where she wanted to be, thanks to the interview’* (IT, HCP, involved).

Facilitators observed that some questions prompted patients to think deeply about their wishes. These included questions about understanding the nature of their illness and about what, at this point in their lives, constituted a good day. Others saw value in the ACTION RC ACP conversations because they noticed how it created an opportunity for patients to make decisions about their own care and encouraged them to share those wishes with their HCP. Facilitators considered the involvement of PRs in the ACTION RC ACP conversations as a key benefit. It provided an opportunity for an open and valuable discussion between the patient and the PR. It could be the first time that a PR became aware of their role and of the wishes of the patient. Facilitators often noticed that PRs experienced a myriad of emotions and a feeling of responsibility, which also became apparent to the patient.

*‘…actually, it was still kind of quite challenging, painful, emotional, to talk through some of those experiences again and revisit. But, but equally, she [the mother] wanted to do it for her daughter, and she did it, but it wasn’t easy for her’* (UK, HCP, not involved).

*‘You saw that they, that was often the very first time that they had thought about it and were so open about it and… so I had a couple like that and well, I found that very rewarding’* (BE, no HCP, not involved).

While facilitators emphasized the importance of the PR’s involvement, some reported that this sometimes complicated the ACTION RC ACP conversation due to the strong influence of the PR. They had to talk to two individuals with different perspectives and emotions and, as such, facilitators concluded that the ACTION RC ACP conversation was an intervention for the PR as well.

Facilitators observed that patients also experienced difficulties with some parts of the ACTION RC ACP conversations. Some patients found it difficult to express themselves or to explore what might happen in the future. Other patients or PRs became emotional. There were also patients who did not seem to understand some of the questions, had difficulty making decisions, or expressed being afraid that they could not change preferences once they were documented. These observations led facilitators to think that participation in an ACTION RC ACP conversation required quite an effort from patients because of the time invested, the emotional effort involved, and the energy required in combination with the time and efforts already needed to undergo their current treatment. Therefore, some facilitators thought that having two ACP conversations on top of patients’ normal care and treatment was too much. Nevertheless, facilitators felt that being challenged to openly and honestly discuss all topics at once could be overwhelming or upsetting for some patients.

*‘I get the impression that in part, it is difficult to understand it [the questions], but I don't know if it is difficult to understand because it is formulated in a certain way, or the patient is put in a very complicated position emotionally.’* (IT, HCP, involved).

### Feeling uncertain and responsible

Despite their observation that many patients positively evaluated the ACTION RC ACP conversation, many facilitators remained uncertain about whether these conversations were the right thing for patients. This feeling was caused by the discomfort facilitators experienced in relation to some parts of the script, the observation that having an ACTION RC ACP conversation was emotionally challenging for both the patient and the PR, and the time and energy it took from patients who were already considerably burdened by their treatment, symptoms and side-effects. In particular, HCPs worried about the patients’ wellbeing. In light of this uncertainty, facilitators reported an increased sense of responsibility for ensuring that the patient derived benefits from the ACTION RC ACP conversation and to safeguard their well-being and coping strategies in dealing with their illness. As one facilitator said:

*‘Time must have meaning, that’s what you feel. So there I feel… I always have patients in that phase, but here I’m more aware of what that conversation is supposed to mean, it must be productive in some way’* (NL, HCP, not involved).

Feeling responsible led facilitators to check on patients’ well-being, also after the ACTION RC ACP conversation had finished, and whether they needed any additional support. Facilitators who were not involved in the regular care of patients missed this opportunity.

*“And I think that hard bit is, we’re used to being able to follow up our patients, and [if] we’re worried and we’re thinking they are distressed, (we can) see them again, you know, it’s very easy to pick up the phone. But, with these patients, you are leaving them potentially quite vulnerable and I think that’s really hard, really hard”* (UK, HCP, not involved).

Facilitators’ feeling of responsibility made them develop goals for themselves. These included the need to keep the patient and the PR emotionally in balance, to safeguard the beneficial effects of the ACTION RC ACP conversations for the patient and to create and maintain a trusting relationship throughout the conversation. The need for working with these goals was reinforced, but made more difficult, by the necessity of following the study protocol, including the script, which could be felt as conflicting with the need to respond sensitively to the perceived needs and preferences of patients.

### Learning process

Over time, many facilitators felt better capable of conducting ACP conversations. They referred to this as a learning process during which they had gained skills and had grown more confident to conduct the ACTION RC ACP conversations

*‘It gets better in time. You have to put in some effort, but eventually it gets easier’* (SI, HCP, not involved).

The initial ACTION RC training constituted the foundation of this learning process. All facilitators highlighted the ACTION RC training as essential to understand and become familiar with the scripts and to improve their communication skills. Facilitators mentioned this had helped them to stay attuned to patients’ needs while performing the ACTION RC ACP conversation according to the script.

*‘I did find it [the training] intensive but, I am really grateful that we received it, this training’* (BE, HCP, involved).

In addition to the training, ‘learning by doing’ was also important. Practising the conversations in conjunction with ongoing coaching on the job by the research team, feedback and reflective conversations with colleague facilitators and members of the research team, and feedback of patients and PRs was mentioned to be indispensable.

Reflective conversations, in particular, addressed difficulties that arose during the conversations and the facilitators’ doubts and uncertainties concerning the balance between the beneficence of the conversation and the – emotional- efforts that were required from patient and PR. This was particularly important because of the facilitators’ increased sense of responsibility for the patients’ coping and well-being and their eagerness to make the conversations valuable for patients.

*‘Yes, I still think the feedback moments are the most important of all, to discuss the difficult cases and find a solution together and to… learn from each other’* (BE, no HCP, not involved).

In addition, facilitators felt more comfortable and confident to continue conducting ACTION RC ACP conversations when patients positively valued aspects of the conversation or when the facilitators themselves identified worthwhile aspects from the patients’ perspective. In addition, ‘learning by doing’ taught facilitators the value of certain communication skills such as the teach-back method (in which patients are asked to repeat in their own words what they understood about the discussed topic). Many facilitators also experienced benefits to their personal and professional development by performing ACTION ACP RC conversations. For example, facilitators became key figures for the patients.

*‘I see this as a very good learning experience for myself as a health care professional. And in a personal sense as well. To be a facilitator is basically a privilege’* (SI, HCP, not involved).

### Thoughts about implementation

A number of facilitators worried about the use of scripted conversations in clinical practice. Some facilitators, in particular those from the UK, stressed that the ACTION RC ACP conversations should not simply become a kind of tick box exercise after being implemented. They emphasised the importance of skilled communication and underlined the need for advanced communication skills to deliver ACTION RC ACP conversations effectively and safely and the need to practice in order to become skilled in the art of these conversations. Refining their skills had enabled them to work with the script, and concurrently to reflect upon the non-verbal communication of the patient and the PR:

*‘And that’s my worry, I think, is that the risk is with the guide and the script, that people will just follow it, maybe not pick up on those cues’* (UK, HCP, not involved).

The question whether HCPs who are already involved in patient care should also take up the role of facilitator set the facilitators thinking. Some indicated that it might be better if facilitators were a part of the medical team enabling them to be informed about the patients’ situation and to build on existing relationships.

*‘An existing relationship of trust allows them [patients] to open up about certain subjects and I don't know if they would do this or how they could do this with a stranger in an unfamiliar environment’* (IT, HCP, involved).

In contrast, others felt that it was desirable not to have prior knowledge of the patient to safeguard the openness of the conversation, and that not having a pre-existing relationship also meant that no dilemmas would arise as a result of their other roles as nurses or doctors.

*‘Well you can say, at least you wouldn’t have any preconceived opinions. No, you don’t have any’* (DK, HCP, not involved).

## Discussion

This study of facilitators delivering an ACP intervention revealed that the intervention was supportive to conduct ACP conversations as well as challenging. Facilitators learned that addressing topics that made patients think and discuss their current and future situation and preferences often resulted in meaningful moments during the conversation. In addition, they felt that patients and PRs often positively evaluated the conversation. Concurrently, the use of a scripted approach in a study context forced them to address topics and to ask questions in a way that was very different to their usual approach. Facilitators felt uncomfortable when they felt that this scripted approach threatened rapport with the patient and PR and required considerable –emotional– engagement from patients already managing the considerable demands imposed by serious illness and its treatment. Driven by some uncertainty about whether these conversations were experienced as beneficial by the patient and their PRs, facilitators felt responsible for ensuring that this was the case. Facilitators emphasized this was a matter of ‘learning by doing’, supported by reflective conversations and coaching on the job.

Previous studies on HCPs’ perspectives about carrying out ACP conversations show that HCPs fear taking away the patients’ hope or that, notwithstanding the potential benefits of ACP, the conversations will leave the patient in an emotionally unbalanced state [4–8]. Facilitators in our study also felt the ethical dilemma between beneficence and non-maleficence. To illustrate, HCPs initiated ACP and promoted the benefits of ACP, but at the same time they felt a duty not to harm the patient and to protect potentially vulnerable patients. The findings suggest three aspects that encouraged facilitators in performing the conversations.

Firstly, our study revealed that facilitators went through a learning process during which they noticed that patients actually responded well to questions that they had anticipated would prove difficult. In addition, they learned how to work with the script. These findings indicate that becoming experienced gave HCPs self-confidence in conducting ACP conversations and to asked ACP-related topics they would usually not have asked to prevent emotional disruption or harming the patients’ coping strategy.

Secondly, the participants in this focus group study mentioned that facilitators need to be highly skilled and stressed in particular the need for good communication skills in order to balance working with the script and remaining attuned to the patient’s needs. This is in line with earlier studies that described a lack of communication techniques as a barrier to undertaking ACP conversations [4–9] and that a skilled facilitator might be the critical link to an effective ACP conversation [23–25]. It is interesting that despite the variation in the facilitators’ professional roles and background, none considered themselves to be lacking competence as a facilitator, though some were more experienced and confident in conducting the ACTION RC ACP conversations than others. The combination of the training, ‘learning by doing’ and reflective conversations (including discussion of ethical problems) seems critical to becoming a skilled facilitator. Still, more research is needed, especially from patients’ perspectives, on whether facilitators need clinical or palliative care skills.

Lastly, facilitators in this focus group study described that patients appeared to be grateful for the opportunity to talk about their preferences for future care despite moments of emotional distress. Based on this, it could be argued that emotions expressed during an ACP conversation are a part of the patients’ process of coping with illness. Therefore, HCPs need not label expressed emotions directly as negative and need not consider these emotions as an expression of burden for the patient. To be able to respond carefully to the emotions expressed by patients, facilitators need advanced communications skills [26].

The facilitators thought differently about whether a facilitator should be involved in regular patient care in order to perform high quality ACP conversations. Although Briggs (2004) reported that facilitators should have an understanding of the patient’s disease and its progression, it is not specified whether they should also be involved in regular care for the patient [27]. In the current study, 13 facilitators were involved in the care for patients with whom they had the ACTION RC ACP conversation. Some facilitators argued that being able to build on an existing trusting relationship made them feel more comfortable in asking ACP-related questions. In addition, they stressed the possibility of following-up the patient after the ACP conversation. In contrast, other facilitators mentioned the importance of having a conversation without any knowledge or preconceptions in advance, which may open up the opportunity to really explore the patient’s perspective. Our results showed pros and cons regarding the involvement of facilitators in the regular care for patients. The optimal way forward might also be influenced by the patients’ personal preference to know or not know the facilitator. Therefore, more research is needed to understand in which situation it is helpful for the conversation to be conducted by a facilitator who is already involved in the care for patients or by an independent person.

### Strengths and limitations

Some strengths and limitations of this study have to be taken into account. Firstly, when implementing a new complex intervention, time and experience are necessary to ensure that it is delivered effectively. Although on average facilitators in the study had completed ACP conversations with six patients, this might not have been sufficient for them to achieve proficiency. Secondly, this study was undertaken across six countries. For the purpose of analysis, the focus group transcripts were translated into English as a common language. Some information or nuance might have been lost in translation, which is an issue in all international studies. However, by using the summaries made by each local team and by validating the results with the researchers of each country, we believe that we took sufficient measures to mitigate these losses. Thirdly, some moderators knew the participants before the start of the focus group. This could have influenced the level of openness of the participants. Finally, it should be noted that patients who were willing to be included in the ACTION trial might have self-selected as being receptive to, and ready to discuss, ACP. This might well have influenced the nature of the RC ACP conversations, thus leading the facilitators to have evaluated the conversations more positively.

## Conclusion

Facilitators experienced positive aspects of the ACTION RC ACP conversation as well as challenges. They indicated the importance of support and training to build confidence and becoming skilled in delivering ACP conversations. In particular, support is needed to address difficult topics and ask confronting questions that proved to be of value for patients, but which tended, in practice, to be avoided. Facilitators felt that aspects of the conversations were meaningful to patients and PRs, but also questioned the efforts it took from patients and PRs.

## Supplementary information

**Additional file 1.** The ACTION trial.

**Additional file 2.** The ACTION Respecting Choice Advance Care Planning intervention.

## Data Availability

The datasets generated and/or analysed during the current study are not publicly available, but are available from the corresponding author on reasonable request.

## Abbreviations

ACP: Advance Care Planning
HCP: Health Care Professional
PR: Personal Representative
RC: Respecting Choices

## References

[1] Brinkman-Stoppelenburg A, Rietjens JA, van der Heide A (2014). The effects of advance care planning on end-of-life care: A systematic review. Palliat Med.

[2] Houben CHM, Spruit MA, Groenen MTJ, Wouters EFM, Janssen DJA (2014). Efficacy of advance care planning: a systematic review and meta-analysis. J Am Med Dir Assoc.

[3] Rietjens JAC, Sudore RL, Connolly M (2017). Definition and recommendations for advance care planning: an international consensus supported by the european association for palliative care. Lancet Oncol.

[4] Gott M, Gardiner C, Small N (2009). Barriers to advance care planning in chronic obstructive pulmonary disease. Palliat Med.

[5] Mullick A, Martin J, Sallnow L (2013). An introduction to advance care planning in practice. BMJ.

[6] De Vleminck A, Houttekier D, Pardon K (2013). Barriers and facilitators for general practitioners to engage in advance care planning: A systematic review. Scand J Prim Health Care.

[7] Johnson S, Butow P, Kerridge I, Tattersall M (2016). Advance care planning for cancer patients: A systematic review of perceptions and experiences of patients, families, and healthcare providers. Psychooncology.

[8] Pollock Kristian, Wilson Eleanor (2015). Care and communication between health professionals and patients affected by severe or chronic illness in community care settings: a qualitative study of care at the end of life. Health Services and Delivery Research.

[9] Ke LS, Huang X, O'Connor M, Lee S (2015). Nurses’ views regarding implementing advance care planning for older people: A systematic review and synthesis of qualitative studies. J Clin Nurs.

[10] Jabbarian LJ, Zwakman M, van der Heide A (2018). Advance care planning for patients with chronic respiratory diseases: A systematic review of preferences and practices. Thorax.

[11] Royal College of Physicians, National Council for Palliative Care, British Society of Rehabilitation Medicine, British Geriatrics Society, Alzheimer's Society, Royal College of Nursing, Royal College of Psychiatrists, Help the Aged, Royal College of General Practitioners. Advance care planning. Concise guidance to good practice series. 2009;No 12.

[12] Bernacki RE, Block SD (2014). American College of Physicians High Value Care Task Force. Communication about serious illness care goals: A review and synthesis of best practices. JAMA Intern Med.

[13] Peck V, Valiani S, Tanuseputro P (2018). Advance care planning after hospital discharge: Qualitative analysis of facilitators and barriers from patient interviews. BMC Palliat Care.

[14] Hammes BJ, Briggs L (2007). ACP facilitator's manual, 3rd edition, la cross.

[15] MacKenzie Meredith A., Smith-Howell Esther, Bomba Patricia A., Meghani Salimah H. (2017). Respecting Choices and Related Models of Advance Care Planning: A Systematic Review of Published Evidence. American Journal of Hospice and Palliative Medicine®.

[16] Kirchhoff KT, Hammes BJ, Kehl KA, Briggs LA, Brown RL (2010). Effect of a disease-specific planning intervention on surrogate understanding of patient goals for future medical treatment. J Am Geriatr Soc.

[17] Pecanac KE, Repenshek MF, Tennenbaum D, Hammes BJ (2014). Respecting choices(R) and advance directives in a diverse community. J Palliat Med.

[18] Hammes BJ, Rooney BL, Gundrum JD (2010). A comparative, retrospective, observational study of the prevalence, availability, and specificity of advance care plans in a county that implemented an advance care planning microsystem. J Am Geriatr Soc.

[19] Rietjens JA, Korfage IJ, Dunleavy L (2016). Advance care planning--a multi-centre cluster randomised clinical trial: The research protocol of the ACTION study. BMC Cancer.

[20] Dierckx de Casterle B, Gastmans C, Bryon E, Denier Y (2012). QUAGOL: A guide for qualitative data analysis. Int J Nurs Stud.

[21] Tong A, Sainsbury P, Craig J (2007). Consolidated criteria for reporting qualitative research (COREQ): a 32-item checklist for interviews and focus groups. Int J Qual Health Care.

[22] Holloway I. A-Z of qualitative research in healthcare. 2nd ed. United Kingdom: Wiley-Blackwell; 2008.

[23] Detering K, Silvester W, Corke C (2014). Teaching general practitioners and doctors-in-training to discuss advance care planning: Evaluation of a brief multimodality education programme. BMJ Support Palliat Care.

[24] Bristowe K, Horsley HL, Shepherd K (2015). Thinking ahead--the need for early advance care planning for people on haemodialysis: A qualitative interview study. Palliat Med.

[25] Briggs LA, Kirchhoff KT, Hammes BJ, Song MK, Colvin ER (2004). Patient-centered advance care planning in special patient populations: a pilot study. J Prof Nurs.

[26] Vanderhaeghen Birgit, Van Beek Karen, De Pril Mieke, Bossuyt Inge, Menten Johan, Rober Peter (2018). What do hospitalists experience as barriers and helpful factors for having ACP conversations? A systematic qualitative evidence synthesis. Perspectives in Public Health.

[27] Briggs L (2004). Shifting the focus of advance care planning: Using an in-depth interview to build and strengthen relationships. J Palliat Med.

