# Supplementary material for: Trained facilitators’ experiences with structured advance care planning conversations in oncology: an international focus group study within the ACTION trial
Source: BMC Cancer. 2019 Oct 31;19:1026. doi: 10.1186/s12885-019-6170-7 (PMC6822448; doi:10.1186/s12885-019-6170-7)
Supplement: Supplementary file 1 — Additional file 1. The ACTION trial. [file 12885_2019_6170_MOESM1_ESM.docx]

**Additional file 1.** The ACTION trial

The primary objective of the ACTION trial is to test the effectiveness of an adapted version of the Respecting Choices (RC) ACP programme among patients affected by advanced lung (small cell – extensive disease/ stage III of IV and non-small cell – stage III of IV) and colorectal cancer (stage IV of metachronous metastases) in a cluster randomised design. Twenty-two hospitals in six European countries —Belgium (BE), Denmark (DK), Italy (IT), the Netherlands (NL), Slovenia (SI) and the United Kingdom (UK)— were randomised in the intervention arm (ACTION RC ACP programme) or control arm (care as usual). In total, 1360 patients will be included (Trial Number: ISRCTN63110516)
